# CD44 in Ovarian Cancer Progression and Therapy Resistance—A Critical Role for STAT3

**DOI:** 10.3389/fonc.2020.589601

**Published:** 2020-12-01

**Authors:** Antons Martincuks, Pei-Chuan Li, Qianqian Zhao, Chunyan Zhang, Yi-Jia Li, Hua Yu, Lorna Rodriguez-Rodriguez

**Affiliations:** ^1^ Department of Immuno-Oncology, Beckman Research Institute, City of Hope Comprehensive Cancer Center, Duarte, CA, United States; ^2^ Department of Surgery, City of Hope Comprehensive Cancer Center, Duarte, CA, United States

**Keywords:** CD44, STAT3, chemoresistance, tumor microenvironment, ovarian cancer, tumor progression, angiogenesis, immunosuppression

## Abstract

Despite significant progress in cancer therapy over the last decades, ovarian cancer remains the most lethal gynecologic malignancy worldwide with the five-year overall survival rate less than 30% due to frequent disease recurrence and chemoresistance. CD44 is a non-kinase transmembrane receptor that has been linked to cancer metastatic progression, cancer stem cell maintenance, and chemoresistance development via multiple mechanisms across many cancers, including ovarian, and represents a promising therapeutic target for ovarian cancer treatment. Moreover, CD44-mediated signaling interacts with other well-known pro-tumorigenic pathways and oncogenes during cancer development, such as signal transducer and activator of transcription 3 (STAT3). Given that both CD44 and STAT3 are strongly implicated in the metastatic progression and chemoresistance of ovarian tumors, this review summarizes currently available evidence about functional crosstalk between CD44 and STAT3 in human malignancies with an emphasis on ovarian cancer. In addition to the role of tumor cell-intrinsic CD44 and STAT3 interaction in driving cancer progression and metastasis, we discuss how CD44 and STAT3 support the pro-tumorigenic tumor microenvironment and promote tumor angiogenesis, immunosuppression, and cancer metabolic reprogramming in favor of cancer progression. Finally, we review the current state of therapeutic CD44 targeting and propose superior treatment possibilities for ovarian cancer.

## Introduction

Ovarian cancer is a global problem and is the most common cause of death in female patients with gynecological tumors in the USA, ranking number five in female cancer deaths (1). The etiology of ovarian cancer remains elusive and the main reason for high mortality rates is the lack of effective screening strategies that results in disease diagnosis at an advanced stage (2). Standard of care for newly diagnosed ovarian cancer patients typically consists of debulking surgery and platinum-based chemotherapy. However, despite current treatment progress in recent years, the prognosis remains poor with five-year survival rates of < 30% depending on geographical location (3, 4). Advanced disease has a recurrence rate of > 80% and demonstrates significant heterogeneity of tumor cells and underlying molecular mechanisms. This leads to resistance to standard chemotherapy regimens (5–9). Given the poor prognosis, limited early screening options, and high prevalence of ovarian cancer chemoresistance, it is vital to identify predictive molecular markers of survival and therapy resistance and identify novel therapeutic targets. CD44, a cell surface protein, has been previously shown to play an important role in cancer stem cell (CSC) function and driving the progression of several tumor types, including ovarian (10–12). There is ample evidence for CD44 expression and signaling in the development of cancer therapy resistance and several publications in various tumor models demonstrate functional crosstalk between CD44 and STAT3 (signal transducer and activator of transcription 3). Here, we explore CD44 function in the context of promoting ovarian cancer therapy resistance, review relevant literature evidence linking CD44 and STAT3 cooperation in tumor progression, and discuss different therapeutic strategies to target CD44 alone or in combination with other molecular targets to improve clinical outcomes in ovarian cancer patients.

## CD44 Structure and Function

**Figure 1:**
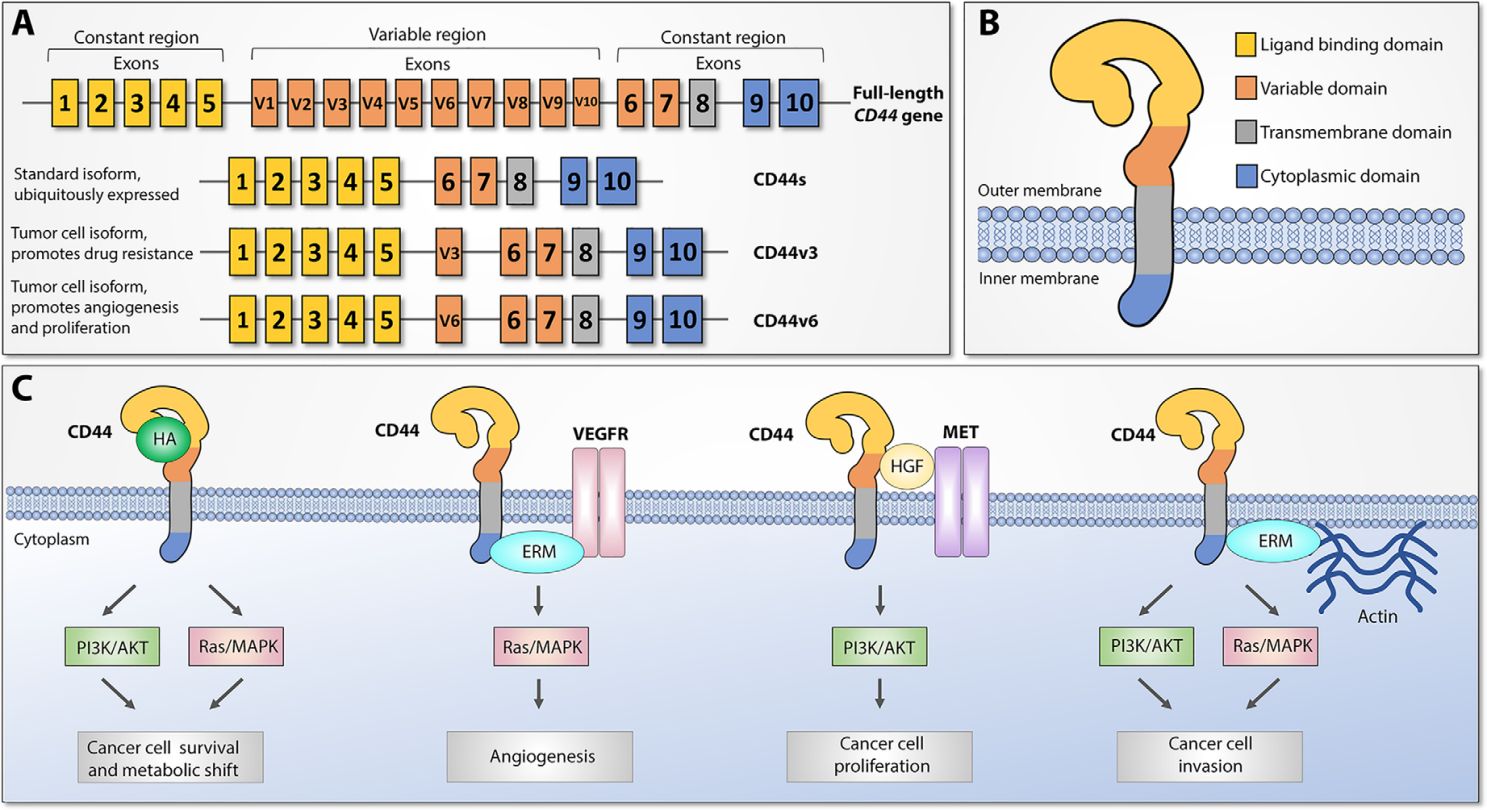
CD44 structure and downstream signaling pathways, adapted from (12). **(A)** Top: CD44 gene structure. CD44 full-length pre-mRNA consists of 20 (mice) or 19 (human) exons, the first and last 5 of which are constant and 9-10 exons in the middle are variable (v) exons regulated by alternative splicing. Bottom: standard (CD44s) and most widely studied cancer-associated alternatively spliced variant isoforms (CD44v3 and CD44v6). Exon coloring parallels corresponding protein domains. **(B)** CD44 protein structure. Four main regions of the CD44 protein are presented with exon matching colors: constant extracellular ligand binding domain, variable extracellular domain, constant transmembrane domain, and cytoplasmic domain. **(C)** Main CD44-mediated downstream signaling pathways. Canonical CD44 activation relies on extracellular ligand stimulation, such as HA and subsequent PI3K and MAPK pathway activation, which leads to cancer cell metabolic shift and resistance to apoptotic stimuli. Via intracellular ERM protein recruitment, the cytoplasmic tail of CD44 can either interact with VEGFR and support tumor angiogenesis or promote cytoskeletal changes and promote cancer cell invasion. Additionally, CD44 may act as a coreceptor for several receptor tyrosine kinases, such as Met, to facilitate cancer progression. ERM: ezrin, radixin, and moesin. VEGFR: Vascular endothelial growth factor receptor.

CD44 (also referred to as HCAM, Hermes antigen or lymphocyte homing receptor) is a cell surface glycoprotein that mediates cellular responses to the microenvironment and is involved in a variety of intracellular processes including proliferation, cell survival, motility, and differentiation (13). CD44 was first identified and cloned in 1989 (14–16) and represents a polymorphic group of surface proteins that range from 80 to 200 kDa in size (12, 17, 18). All CD44 proteins are encoded by a single highly conserved gene that is comprised of 20 exons in mouse and 19 exons in human genome. The size heterogeneity of CD44 gene products is explained by either different post-translational modifications (N- and O-glycosylation) or by alternative splicing, which gives rise to many CD44 isoforms (19–21). The first and last five exons are always expressed and encode the smallest standard isoform (CD44s) with the central ten variable exons spliced out (**Figure 1A**). The variable middle nine exons can be alternatively spliced and assembled in different combinations, referred to as CD44 variant (CD44v) isoforms. Standard CD44 protein is comprised of four main domains: extracellular ligand binding, variable, transmembrane, and cytoplasmic (**Figure 1B**). Variable exon splicing mainly affects the structure of extracellular membrane-proximal regions of CD44 and up to ten different CD44v isoforms have been described (11–13). CD44s is present on the surface of most vertebrate cells and is typically described in the context of hematopoiesis (22).

CD44 proteins orchestrate diverse molecular functions in three main ways (**Figure 1C**). First, CD44 receptors serve as a non-kinase transmembrane receptor that can actively or passively bind ligands, such as hyaluronic acid (HA), ostepontin (OPN), collagen, fibrin, and others, and mediate interaction with the extracellular microenvironment (ECM). HA interaction leads to CD44 conformational changes that allow adaptor molecule binding to the intracellular cytoplasmic tail, which leads to activation of various signaling pathways, such as Ras/MAPK and PI3K/Akt, and facilitates cell proliferation, adhesion, and migration. Second, both CD44s and CD44v splice variants have been shown to act as a co-receptor to receptor tyrosine kinases (RTK), such as Met or the Epidermal Growth Factor (EGF) family ErbB, as well as stabilize other RTK complexes, such as receptors for vascular endothelial growth factor (VEGFR), which under pathological conditions leads to tumor progression, metastasis, and angiogenesis. Third, CD44 mediates cytoskeletal changes through interactions with actin-binding ERM (ezrin, radixin, and moesin) proteins, which are necessary for cellular movement and inducing either proliferation- and metastasis-promoting signaling or proliferation- and metastasis-inhibitory molecular complexes, depending on extracellular signals. These findings indicate that CD44 proteins have highly conserved widespread biological functions, and under pathological conditions they may play an important role in promoting cancer progression, metastasis, and resistance to therapy (13).

## CD44 in Ovarian Cancer Progression and Therapy Resistance

Activated CD44 proteins interact with several intracellular signaling networks that support the oncogenic properties of tumor cells and drive cancer progression, metastasis, and therapy resistance across various cancer models (11, 12, 19, 23). Expression of different CD44 isoforms positively correlates with poor clinical outcome in various cancers, such as breast (24–26), colon (27), lung (28), bone (29), pancreatic (30), colorectal (31), bladder (32), gastric (33), and head and neck squamous cell carcinomas (34), as well as leukemias (35) and lymphomas (36). In line with these observations, CD44 has been increasingly implicated in the pathogenesis and poor clinical outcomes of ovarian cancer patients as well (37).

### CD44 Expression in Ovarian Cancer Progression and Metastasis

CD44 expression has been found in most epithelial ovarian carcinoma (EOC) tumors and higher CD44 levels correlate with more advanced disease stage (37, 38). Several publications demonstrate the association of CD44 with poor prognosis in EOC patients (37, 39–42), including a recent systematic meta-analysis of 18 studies consisting of more than 2000 ovarian cancer patients, which showed a significant correlation between CD44 expression and poor 5-year overall survival (43). This demonstrates that CD44 levels are an effective marker for diagnosis and prediction of clinical outcomes. Importantly, upregulation of CD44 in ovarian cancer has been shown to be strongly associated with the occurrence of metastasis and disease relapse (44, 45). Specifically, Gao et al. analyzed patient-matched primary, metastatic, and recurrent tumor samples from 26 ovarian cancer patients and showed higher CD44 expression in metastatic and relapsed tumor tissues compared to patient-matched primary tumors, while at the same time CD44 knockdown significantly reduced proliferation and invasion capability of ovarian cancer cells *in vitro* (44). Additionally, several studies reported that CD44 expression was correlated with the epithelial to mesenchymal transition (EMT) phenotype *in vitro* and in patient samples. EMT is necessary for cells to detach from the epithelial layer and invade secondary sites to form metastases (46, 47). This study further confirms an important role for CD44 in ovarian cancer progression.

### CD44 in Ovarian Cancer Stemness and Chemoresistance

CD44 surface expression has been linked to cancer stem cells (CSCs), in ovarian and other cancer models (11, 48, 49), and most studies about the role of CD44 in ovarian cancer progression emphasize the connection between CD44 and CSC maintenance. CSCs are a regenerative tumor cell sub-population that has attained stem cell-like properties, which allows these cells to drive tumor recurrence and chemoresistance. CD44 has been implicated as one of the potential biomarkers of ovarian CSCs (48, 50, 51). CD44-positive (CD44+) ovarian tumor cell subpopulations have been shown to express stem cell markers and are able to initiate tumorigenesis and promote disease recurrence by recapitulating the original tumor (52, 53). More importantly in the context of this review, CD44+ stem-like cells have been shown to be markedly resistant to paclitaxel and platinum treatment, two standard front-line therapeutics against ovarian tumors (54). CD44+ and CD44-negative (CD44-) ovarian cancer cell fractions have been described as Type I chemoresistant and Type II chemosensitive EOC cells, respectively. Only cells expressing CD44 on the surface persist after chemotherapy treatment and are able to rebuild the tumor afterwards (55). Clinical studies of chemotherapy sensitive or resistant EOC patients show significant correlation between CD44 upregulation and chemotherapeutic drug resistance (56, 57), and numerous reports further confirm the role of CD44 in promoting chemoresistance in primary ovarian tumors, spheroids and ascites, as well as human ovarian cancer cell lines *in vitro* (51, 53, 54, 58). In line with these observations, drug resistant ovarian cancer cells show higher CD44 levels *in vitro* (44), while genetic overexpression of CD44s induces stem-like properties and chemoresistance in xenograft mouse models and ovarian cancer cell lines (44, 59). At the same time, CD44 knockdown significantly enhances paclitaxel, cisplatin, and doxorubicin sensitivity in ovarian cancer cells (44, 47, 60). Collectively, these findings indicate a pivotal role for CD44 in therapy resistance development, which is currently a major challenge in the treatment of ovarian cancer patients. Identifying the precise molecular mechanisms of CD44 in promoting stem cell-like features and drug resistance would be highly advantageous in order to discover novel treatment strategies. However, due to the large number of signaling networks modulated by CD44, it is important to define relevant CD44 molecular interaction partners that aid in the promotion of ovarian tumor resistance to front-line therapeutics in clinic. Several studies identified STAT3 as an important interaction partner for CD44 in promoting tumor properties across various cancer models, including ovarian. Therefore, here, we evaluate all existing evidence indicating functional CD44 and STAT3 cooperation in the context of tumor metastatic progression, therapy resistance and immunosuppression with the focus on ovarian cancer. We will focus on tumor-specific and intracellular cross-regulation first, after which we will review and the ample evidence for CD44 and STAT3 crosstalk within the tumor microenvironment (TME) on the levels of new blood vessel formation, cancer-associated fibroblast activation and immunoregulatory cell recruitment. Finally, we will discuss CD44 and STAT3 involvement in cancer-driven metabolism switches and summarize currently available data on CD44 therapeutic targeting alone or in combination with STAT3, highlighting promising therapeutic opportunities for the future.

## CD44 and STAT3 Crosstalk in Cancer

STAT3 is a pleiotropic transcription factor that belongs to a family of STAT transcription factors and is involved in the regulation of numerous intracellular processes. Among the most well-known extracellular STAT3 activators are a large number of cytokines, chemokines and growth factors, as well as many tyrosine kinases that are upregulated in cancer. Traditional intracellular STAT3 activation involves tyrosine 705 phosphorylation by Janus kinases (e.g. JAK1, JAK2) or other tyrosine kinases as well as post translational modifications, such as acetylation, followed by nuclear translocation, where STAT3 binds to DNA and regulates the expression of target genes. Activated STAT3 is a critical contributor of cancer cell survival and proliferation, and tumor invasion, metastasis. STAT3 is also well-known to induce immunosuppression to promote tumor progression (61–63). Over the years, STAT3 has been shown to interact with several other transcription factors and signaling pathways at multiple levels to support cancer progression. One of such STAT3 interacting molecules is CD44. As with CD44, STAT3 signaling has been frequently implicated in ovarian cancer metastasis, therapy resistance, and CSC maintenance (64, 65). STAT3 transcriptional activity has been demonstrated to drive the migration and invasiveness of ovarian cancer (66–68). Specifically, activated STAT3 has been shown to induce matrix metalloproteinase 2 and 9 (MMP-2, MMP-9) expression in ovarian cancer models (68, 69), which are important factors involved in the degradation of extracellular matrix necessary for tumor invasion and metastasis (70). Moreover, STAT3 signaling promotes ovarian cancer resistance to cisplatin and paclitaxel, which can be reversed by either genetic or pharmacological STAT3 inhibition (71–74). Given that CD44 upregulation in patient samples and cell lines is also strongly correlated with an acquired resistance against the first line ovarian cancer therapeutics (53–57), we hypothesize that functional interaction between CD44 and STAT3 may be one of the driving mechanisms of ovarian cancer progression and therapy resistance, which deserves further attention. Below, we summarize currently existing evidence of functional and direct collaborations between CD44 and STAT3 signaling pathways across various cancer models.

During the last decade, several studies have shown that CD44 and STAT3 cooperate in cancer promotion. In the context of breast cancer, STAT3 signaling has been shown to be required for maintenance of self-renewal and growth of CD44+ breast CSCs (75). Additionally, siRNA-mediated STAT3 inhibition has been shown to reverse tamoxifen resistance of CD44+ breast CSCs, indicating a central role for STAT3 not only in CSC maintenance but also in therapeutic resistance development (76). Furthermore, numerous studies indicate that CD44 and STAT3 regulate each other’s expression and activity. In prostate cancer, CD44 expression significantly correlates with IL-6, a STAT3-activating cytokine, and IL-6 or STAT3 inhibition both decrease CD44 and EMT-related protein levels in cancer cell lines (77, 78). In agreement with these observations, monoclonal antibodies targeting the CD44s isoform reduce CSC percentage in cultured pancreatic cancer cells and in xenograft mouse models, along with downregulating STAT3 levels and STAT3-mediated target gene expression (79). In breast and urinary bladder cancer cell lines, CD44 knockdown inhibits cell invasion and tumorigenicity via STAT3 phosphorylation blockade, while anti-CD44 blocking antibody treatment downregulates STAT3 levels in rat atrial fibroblasts, suggesting that CD44 can regulate both STAT3 expression and activation (80–83). In turn, well known STAT3 activators IL-6 (84) and IGF-1 (85) have been shown to significantly induce CD44 promoter activity in pancreatic tumor cells (12), while in hepatocytes several putative STAT3 binding sites have been described within the CD44 promoter, demonstrating that STAT3 can directly induce CD44 expression (86). In line with these observations, STAT3 blockade via siRNA or specific inhibitors has been reported to significantly decrease CD44 expression in breast, prostate, nasopharyngeal, and gastric carcinoma models (77, 78, 80, 81, 87, 88). Collectively, these results show that CD44 and STAT3 can regulate each other’s expression and cooperate across different tumor types to drive cancer invasion, metastasis, disease recurrence, and chemoresistance. **Table 1** summarizes the data from published studies in which different CD44 isoforms were shown to cooperate with STAT3 in different cancer models. (76–79, 81, 83, 86, 87, 89–92)

**Table 1:** Reported cooperation between CD44 and STAT3 in different cancer models.

Ref.	CD44 isoforms	Cooperation mechanism	Biological implications	Cancer type
(79)	CD44s	CD44 activates STAT3 via Nanog	Tumor growth metastasis low survival rate, self-renewal (CD44+CD24-)	Pancreatic cancer
(77)	CD44s	IL-6/STAT3 signaling promotes CD44 expression	Increased colony formation, metastasis, tumor aggressiveness	Prostate cancer
(89) (76) (81)	CD44s	CD44 activates STAT3 via Nanog IL6/STAT3 signaling promotes CD44 expression CD44 induces hTERT via STAT3/NF-kB complex	Tumor progression, chemoresistance, stemness, migration and metastasis	Breast cancer Ovarian cancer
(86)	CD44s	IL6/STAT3 signaling promotes CD44 expression	Tumor progression, tumor growth	Liver Cancer
(90)	CD44, not specified	Nuclear CD44 binds acetylated STAT3 and promotes target gene expression	Cancer cell reprogramming, tumor progression, metastasis	Colon cancer
(91)	CD44s	Concurrent expression of CD44 and STAT3 in patients	Advanced tumor stage, poor survival	Clear renal cell carcinoma
(83)	CD44v3	CD44 activates STAT3 along with PI3K and ERK signaling cascades	Tumor survival and progression	Bladder cancer
(87) (92)	CD44v6	IL6/STAT3 signaling promotes CD44 expression CD44 expression increases cell survival via STAT3 and P38	Tumor growth, invasion, metastasis, progression and chemoresistance	Gastric Carcinoma
(78)	CD44, not specified	STAT3 signaling promotes CD44 expression	Resistance to anoikis, invasiveness, metastasis	Nasopharyngeal carcinoma

**Figure 2:**
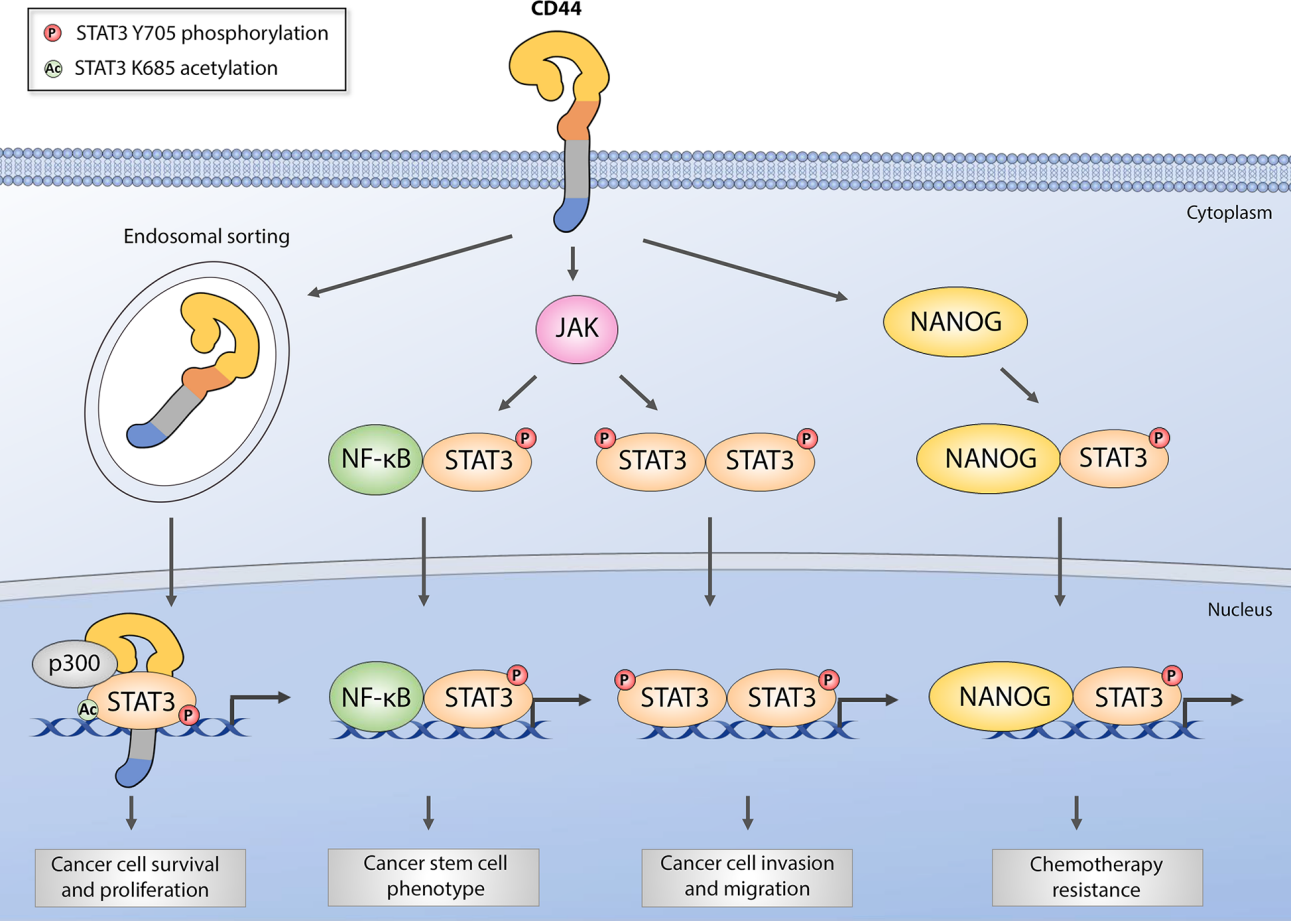
Graphical representation of intracellular CD44 and STAT3 interactions in various cancer models. Full-length CD44 can translocate to the nucleus via endosomal sorting and form nuclear complexes with histone acetyltransferase p300 and STAT3, which supports STAT3 phosphorylation and acetylation and subsequent pro-tumorigenic gene induction. Membrane-bound CD44 isoforms are capable of inducing canonical JAK-mediated STAT3 activation by critical tyrosine 705 phosphorylation, leading to either classical activated STAT3 dimer formation and target gene induction, or formation of nuclear NF-kB and STAT3 complex critical for cancer stem cell gene expression. Finally, HA-mediated CD44 activation has been shown to promote cytoplasmic NANOG/STAT3 complex formation involved in multiple drug resistance gene upregulation and chemoresistance.

Additionally, several direct intracellular interactions between CD44 and STAT3 have been reported across different cancer models. In breast cancer cells, transmembrane CD44 isoforms have been shown to either physically associate directly with JAK2 and STAT3 to induce conventional STAT3 oncogenic signaling (80) or activate STAT3 in the cytoplasm to form a complex with NF-kB p65 subunit in order to initiate the transcription of human telomerase reverse transcriptase (hTERT), which further leads to EMT and breast CSC phenotype maintenance (81). Next, across various cancer models, full-length CD44 has been shown to translocate to the nucleus, physically bind to nuclear STAT3 and p300 acetyltransferase, and promote STAT3 acetylation at lysine 685, which elicits cell proliferation and stem cell-like phenotype in colon, gastric, and lung cancer cell lines (90, 93). Co-IP experiments showed that the coiled-coil domain of STAT3 and C-terminal domain of CD44 are important for CD44/STAT3 complex formation. Finally, Bourguignon and colleagues showed that HA-induced CD44 activation promoted STAT3/Nanog complex formation, which led to the upregulation of multidrug resistance marker MDR1 in breast, ovarian, and head and neck squamous cell cancers (89, 94). In line with these observations, we have previously shown that CD44 promotes multidrug resistance and MDR1 stabilization in ovarian cancer cells (95, 96), while STAT3 signaling has been shown to induce MDR1 expression in ovarian, lung and bone cancers, as well as a myeloid leukemia model (97–100). All reported CD44 and STAT3 intracellular interactions are graphically summarized in **Figure 2**.

Taken together, these findings indicate that the promotion of metastasis, stem cell-like phenotype, and chemoresistance involves intracellular cooperation between CD44 and STAT3 on a molecular level across numerous cancer models, including ovarian. Therefore, CD44 and STAT3 crosstalk deserves further investigation to identify promising novel strategies to sensitize ovarian tumors to treatment and prevent disease relapse.

## CD44 and STAT3 in Ovarian Tumor Microenvironment

Cancers encompass not only masses of malignant tissue but also recruit and exploit different types of non-transformed cells. The tumor microenvironment (TME) refers to the surrounding stroma where tumor grows and complex biological interactions between malignant and non-transformed cells occur. Non-malignant cells present within the TME often demonstrate tumor-promoting functions and further facilitate disease progression, immunosuppression, and metastasis (101–103). Functional interaction between CD44 and STAT3 can also occur at different levels of the TME, discussed in detail below, and encompass tumor endothelial cells, fibroblasts, and cells of the immune system, such as myeloid-derived suppressor cells, macrophages, and regulatory T and B cells.

### CD44 and STAT3 in Endothelial Cells for Tumor Angiogenesis

New blood vessel formation is required for blood supply to satisfy oxygen and nutrient demands of tumor tissues. This is achieved through a process called angiogenesis, where hypoxic conditions stimulate vascular endothelial growth factor (VEGF) secretion and start a multidimensional process regulated by cancer cells in concert with various immune cells, fibroblasts, and other TME cells that results in the growth of new blood vessels, which further supports tumor survival and induction of metastasis (104, 105). At the molecular level, both CD44 and STAT3 have been reported to support tumor angiogenesis in several tumor models. Numerous studies provide evidence of the involvement of endothelial cell-associated HA/CD44 signaling in normal angiogenesis (106–109). In the context of tumor vascularization, epithelial cells from the blood vessels of solid tumors show increased CD44 levels compared with normal tissue samples (110, 111), and CD44 inhibition blocks tumor induced angiogenesis in human melanoma and laryngeal cancer models (112). Moreover, high CD44 expression levels are significantly correlated with increased VEGF and both factors are associated with an adverse prognosis for renal cell carcinoma patients (113). Finally, endothelial CD44 has been shown to be essential for wound healing and vascularization, as well as ovarian tumor angiogenesis *in vivo* (114). Likewise, ample evidence suggests that STAT3 regulates many aspects of tumor angiogenesis at the transcriptional level (115). Our group and others previously demonstrated that STAT3 is an essential mediator of endothelial activation by directly inducing VEGF and HIF1a gene expression (116, 117). On a functional level, STAT3 signaling has been reported to promote angiogenesis in human pancreatic, lung, and breast cancers (118–120) and we have previously shown that STAT3 is a critical regulator of the pro-angiogenic functions of myeloid cells in mice (121). In ovarian cancers, the expression of VEGF and its receptors VEGFR1 and VEGFR2 significantly correlate with pSTAT3 levels in patient samples (122), and STAT3 and VEGF have been shown to reciprocally regulate each other’s expression and activation in EOC models (123, 124).

Furthermore, both CD44 and STAT3 have been shown to crosstalk with matrix metalloproteinases, which are important mediators of tumor angiogenesis (125). We and others have previously demonstrated that CD44 signaling upon HA or OPN treatment stimulated the synthesis of MMP-2 and MMP-9, which facilitated extracellular matrix degradation and subsequent disease progression (126–129). In cancer cell line models CD44+ cells demonstrated significantly elevated MMP-2 and MMP-9 levels compared to CD44- cells, further indicating that CD44 is involved in MMP-2 and MMP-9 expression (130). Apart from that, CD44 also plays an important role in MMP-2 and MMP-9 activation through the binding of proteolytically active MMP-2 and MMP-9 isoforms to the membrane and promoting the cleavage of latent TGF-β, which led to invasion and angiogenesis across various cancer models (131–133). Similarly, STAT3 also promotes the expression of matrix metalloproteinases MMP-2 and MMP-9, as well as basic ﬁbroblast growth factor (bFGF) genes, which are also implicated in new blood vessel formation (118, 134, 135). However, despite both STAT3 and CD44 acting as pivotal regulators of tumor angiogenesis, there is currently no direct evidence of functional crosstalk between STAT3 and CD44 during angiogenesis promotion. One potential indication of CD44 and STAT3 cooperation in new blood vessel formation is an observation made by Wang and colleagues (124). They reported that HA/CD44 signaling promoted epithelial tube and new blood vessel formation via activation of Src that in turn enhanced expression of c-Jun and c-Fos transcription factors. Our laboratory and other groups have previously established Src kinases as important upstream activators of STAT3 during oncogenesis (136–139), while c-Jun and c-Fos have been shown to form a complex with STAT3 that binds to IL6 response elements (140, 141), indicating that CD44 and STAT3 may cooperate in new blood vessel formation. The numerous observations that both CD44 and STAT3 are critically involved in the VEGF pathway and tumor angiogenesis indicate that dual targeting of both factors could be a promising therapeutic target to improve antiangiogenic treatment in ovarian cancer. Given that increased angiogenesis has been shown to be associated with cancer therapy resistance in ovarian cancer ascites (142), targeted inhibition of new blood vessel formation by ovarian tumors will aid in improving therapy outcomes.

### CD44 and STAT3 in Cancer-Associated Fibroblasts

Under normal conditions, resident tissue fibroblasts support tissue integrity and homeostasis. Cancer-associated fibroblasts (CAFs) are an immensely heterogenous stromal cell subpopulation that resides in the TME and hijacks normal physiological functions of fibroblasts to drive solid tumor growth, angiogenesis, and inhibition of anti-tumor immune responses (143, 144). In ovarian cancer, CAFs are prominent components of the ovarian TME and have been shown to support cancer cell proliferation and metastasis by inducing EMT and angiogenesis, as well as immunosuppression and the cancer cell metabolism switch (145). More importantly, CAFs also promote the CSC phenotype and chemoresistance development in ovarian tumor models (145, 146) and several studies have demonstrated that CD44 and STAT3 signaling pathways are involved in CAF-mediated therapy resistance. CD44+ ovarian cancer tumors have been shown to reside near tumor stroma. Our immunohistochemical analysis of CD44 expression in EOC patients indicated CD44 involvement in a functional crosstalk between tumor and surrounding stroma (55, 147). In line with these observations, TGF-β-activated versican expression in CAFs has been shown to induce the remodeling of ECM and ovarian cancer cell invasion by upregulation and binding to CD44 in ovarian cancer cells (148). At the same time, ovarian CAFs have been shown to be a major source of IL-6 in the TME, which activates STAT3 in ovarian tumor cells leading to cell proliferation and invasion (149). CAF-mediated STAT3 activation in ovarian cancer lines also leads to development of cisplatin resistance through the increased expression of antiapoptotic proteins, indicating a prominent role for STAT3 in CAF-induced chemoresistance (150). In the context of ovarian carcinoma, there are no reports analyzing CD44 or STAT3 signaling within CAFs themselves. However, both high CD44 expression and STAT3 activation in CAFs have been reported to maintain CSC phenotype and promote cancer drug resistance in other cancer types (151–155). Additionally, TGF-β, an important mediator of normal fibroblast transition into ovarian CAFs (143, 148), upregulates CD44 and STAT3 in cultured rat atrial fibroblasts to promote atrial fibrosis (82). Therefore, it is reasonable to postulate that CD44 and STAT3 not only mediate CAF-driven ovarian tumor stemness and chemoresistance within tumor cells, but also cooperate within CAFs themselves to support their cancer-promoting phenotype, which requires further investigation.

### CD44 and STAT3 in Myeloid-Derived Suppressor Cells

Myeloid-derived suppressor cells (MDSCs) represent another major component of the TME and are well known in cancer immunology research for their strong immunosuppressive activity. Like CAFs, MDSCs are a heterogenous cell population consisting of myeloid progenitors and immature macrophages, granulocytes, and dendritic cells. Under normal conditions, immature myeloid cells (IMCs) terminally differentiate into specific immune cell subsets via a process called myelopoiesis in order to protect the host from pathological conditions. However, during cancer progression and low-level chronic inflammation, IMCs fail to terminally differentiate and give rise to MDSCs that migrate to peripheral lymphoid organs and accumulate within the TME and tumor tissues to further support cancer progression. MDSCs are phenotypically classified as monocytic (M-MDSCs) or granulocytic and polymorphonuclear (G-MDSC/PMN-MDSC) MDSC populations and their upregulation and function correlate with progression, recurrence, and therapy resistance of many types of human tumors (156–158). Several reports show MDSC involvement in ovarian cancer progression. In two different publications, MDSCs have been shown to drive immunosuppression in the ID8 ovarian cancer mouse model either by directly downregulating cytotoxic CD8+ T cells or by inducing immunoregulatory CD4+CD25+ Treg cell expansion (159, 160). Apart from suppressing anti-tumor immunity, MDSCs have also been demonstrated to drive CSC gene expression, sphere formation, and metastasis of primary human ovarian cancer cells (161). Since numerous studies show that increased numbers of tumor MDSC are a significant and independent predictor of poor survival rates of ovarian cancer patients (161–165), targeted inhibition of MDSCs and underlying molecular mechanisms could be a promising treatment approach.

Both CD44 and STAT3 have been shown to support pro-tumorigenic MDSC functions. Only high CD44-expressing head and neck squamous carcinoma cells can efficiently induce MDSCs and Treg accumulation (166), while physical interaction of peripheral blood monocytes (PBMCs) with CD44 expressed on the surface of activated hepatic stellate cells leads to monocyte trans-differentiation into MDSCs (167). At the same time, STAT3 has been described as the main transcription factor that drives the expansion and function of MDSCs. Compared to naïve IMCs, MDSCs from tumor-bearing mice demonstrate increased levels of STAT3 activation (168), and JAK2/STAT3 pathway plays an essential role in MDSC expansion from hematopoietic progenitor cells. Namely, targeted STAT3 blockade or conditional hematopoietic STAT3 knockout in mice significantly reduces MDSC population and increases T-cell responses, as we and others have previously shown (169, 170). Notably, tumor-induced STAT3 activation in the M-MDSC subpopulation increases CD44 expression in human pancreatic cancer cells and promotes CSC-like properties (171), indicating that STAT3 transcriptional activity in MDSCs functionally interacts and relies on CD44 signaling to promote cancer stemness and immunosuppression. Because MDSCs promote both ovarian cancer cell stemness and accumulation of ascites in ovarian cancer via STAT3 (172), we propose that targeting STAT3 along with CD44 in ovarian tumors may be a rational strategy for blocking MDSC-driven immunosuppression and enhancing the efficacy of conventional ovarian cancer therapy.

### CD44 and STAT3 in Tumor-Associated Macrophages

Tumor-associated macrophages (TAMs) are the main population of immune cells in ovarian tumor stroma, and CD44 and STAT3 both significantly contribute to tumor promoting properties of the ovarian TME (173). TAMs are either mature macrophages that are recruited to the tumor site and surrounding tissues or they can differentiate from M-MDSC already present within the TME (174, 175). TAMs can have both tumor supportive or inhibitory properties, which mainly depends on TAM polarization into either pro-inflammatory antitumor M1 or immunosuppressive, tumor-promoting M2 phenotype (176). In the TME, persistent STAT3 activation has been shown to suppress the M1 phenotype and promote anti-inflammatory M2 polarization of TAMs, which further promotes tumor growth by enhancing angiogenesis, immunosuppression, cancer cell invasion, and metastasis of several cancer models (177–179). More importantly for the scope of this review, tumor-promoting TAMs have been reported to drive ovarian cancer metastasis, stemness, and therapy resistance with the involvement of STAT3 and CD44. In EOC patients, higher M2 TAM accumulation positively correlates with shorter survival rate (180) and STAT3 activators IL-6 and LIF have been shown to drive M2 TAM phenotype switch in ovarian tumors (181). In line with these reports, ascites from EOC, but not from non-EOC patients, induce M2 macrophage polarization through STAT3 activation (182), indicating a central role for STAT3 in M2 TAM expansion in ovarian tumors. Furthermore, TAM interactions with CD44+ ovarian CSCs have been shown to promote ovarian cancer recurrence and multidrug resistance (183), and a recent study showed that not only increased STAT3 signaling within TAMs can induce CD44 expression and CSC-like phenotype in ovarian cancer cells, but high CD44-expressing ovarian CSCs are able to further promote the M2 phenotype through STAT3 activation in macrophages as well, forming a positive feed-forward loop of mutual TAM and CSC activation via CD44/STAT3 cooperation that results in ovarian cancer stemness maintenance and chemoresistance (184). Collectively, these observations support a prominent role for CD44 and STAT3 crosstalk in mediating tumor and TAM interactions within the ovarian TME.

### CD44 and STAT3 in Regulatory T Cells

Regulatory T cell (Treg) accumulation and suppression of antitumor immunity have been shown in ovarian cancer mouse models, and Tregs are linked to poor prognosis for ovarian cancer patients (62, 185–188). Several studies have demonstrated significant accumulation of activated regulatory Tregs in ascites and tumor tissues from ovarian cancer patients compared with normal ovarian tissue (186, 189). These studies demonstrate that Tregs in the malignant ascites are more activated and have a higher proliferation rate compared to blood-derived cells from the same patients. A recent study showed that Treg cells isolated from ovarian tumors display a distinct cell surface phenotype with increased expression of immunosuppressive receptors, such as PD-1, 4-1BB, and ICOS (187). In addition, high expression of FoxP3, a master regulator of the Treg immunosuppressive phenotype, is associated with poor prognosis in ovarian cancer patients (190).

Despite the progress made in studying Treg activation in ovarian cancer, relatively little is known about the underlying molecular mechanisms. Several studies have implicated CD44 involvement, which is frequently expressed and activated in Tregs. CD44 expression is positively correlated with FoxP3 expression and the suppressive function of Tregs (191), while Treg-specific CD44 depletion enhances antitumor immunity (192) and CD44-knockout mice display functionally impaired Tregs (193). STAT3 has also been confirmed as a critical molecular driver for FoxP3 expression and Treg immunosuppressive phenotype in the tumor setting (194, 195). Based on these studies, CD44 and STAT3 have overlapping functions within tumor-associated regulatory T cells. Indeed, while CD44 co-stimulation promotes the expression of FoxP3 and supports Treg function via IL-2, IL-10, and TGF-β production (193), FoxP3 has been shown to act as a co-transcription factor with STAT3 in tumor-induced Tregs to promote immunosuppressive IL-10 production (196), suggesting that functional CD44 and STAT3 cooperation is one of the main molecular mechanisms that drives Treg immunosuppressive actions.

### CD44 and STAT3 in Regulatory B Cells

Regulatory B cells may promote cancer progression, mainly by IL-10 mediated cytotoxic T cell inhibition. Accumulating evidence has indicated that B cells are involved in ovarian cancer progression (197–199). It has been shown that STAT3 is constitutively active in tumor-associated B cells (200). Furthermore, our group previously reported that CD5 in tumor infiltrating B cells binds to IL-6, and through gp130 induces STAT3 activation to promote cancer development (201). Our previous studies also showed that increased B cell infiltration and p-STAT3 expression in omental tissue are associated with poor survival in ovarian cancer patients (202). Finally, functional CD44/STAT3 crosstalk in human immunoregulatory B cells is further highlighted through the observation that CD44 ligation on B-cells significantly upregulates STAT3-activating IL-6 gene expression (203).

**Figure 3:**
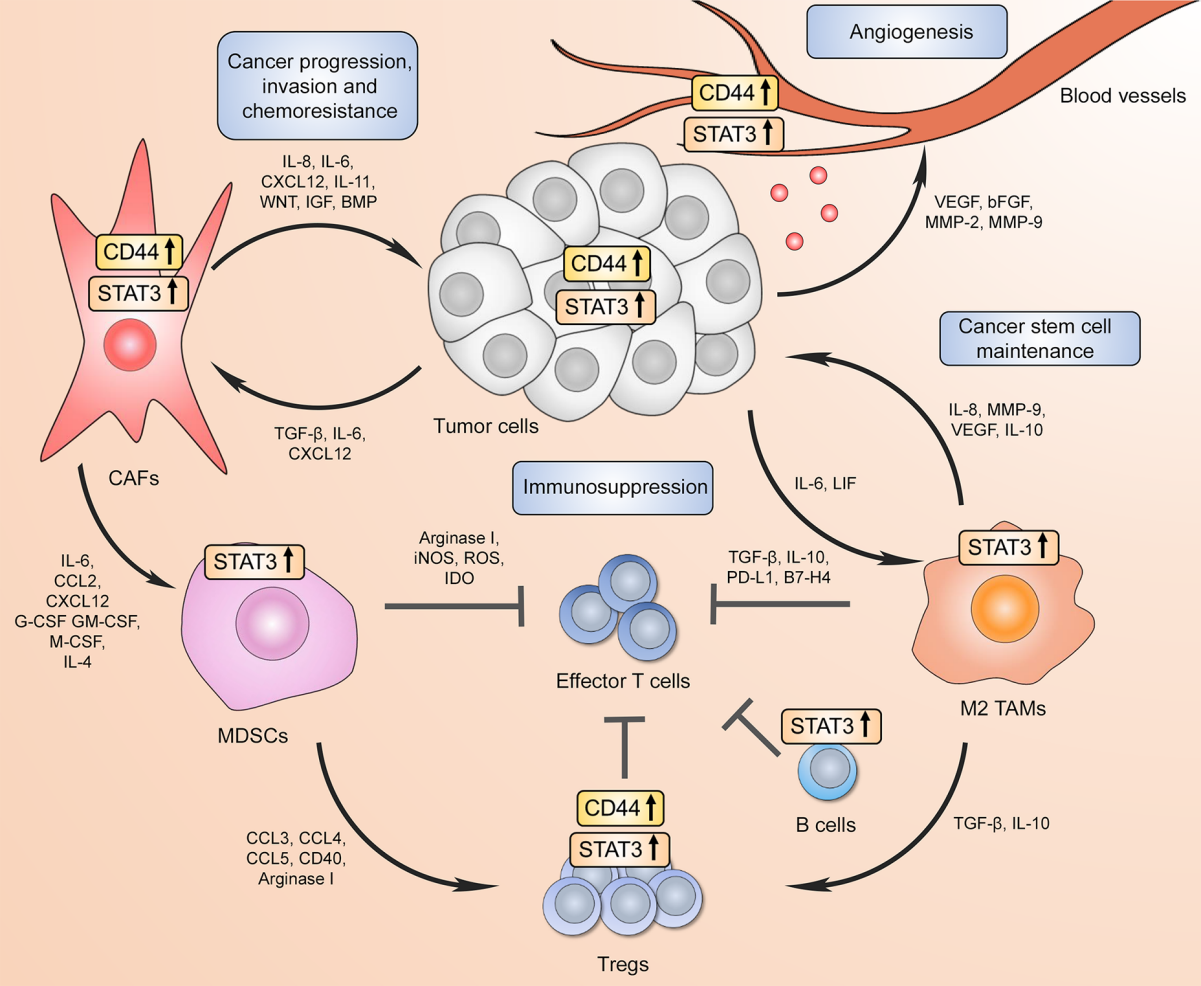
CD44 and STAT3 support the communication between tumor cells and the tumor microenvironment (TME) to drive cancer progression/recurrence, immunosuppression, and chemoresistance. Increased CD44 and STAT3 activity in tumor cells promotes proximal cancer-associated fibroblast differentiation, which in turn further supports tumor progression through pro-tumorigenic factor secretion forming a positive feed-forward loop. In addition, CD44 in both cancer cells and CAFs facilitate the expansion and recruitment of myeloid-derived suppressor cells (MDSCs) that either directly inhibit cytotoxic effector CD8+ T cell function or drive immunoregulatory T cell (Treg) differentiation. Both CD44 and STAT3 contribute to tumor Treg expansion through upregulating FoxP3 expression. High STAT3 activation in B cells also results in immunosuppressive phenotype. At the same time, tumor-associated macrophages (TAMs) in the TME have also been shown to repress effector T cell-mediated anti-tumor immunity through immunosuppressive cytokine production and immune checkpoint expression, which requires STAT3 transcriptional activity. Moreover, increased STAT3 signaling in tumor surrounding TAMs promotes cancer stem cell phenotype, which in turn further drives immunosuppressive macrophage phenotype. Finally, CD44 and STAT3 signaling in both tumor and endothelial cells contributes to new blood vessel formation via angiogenic factor expression.

Taken together, these findings demonstrate that the ovarian TME is a complex multicomponent system that is dynamically supported by different cell types and a variety of underlying molecular mechanisms, including CD44 and STAT3 signaling pathways. The summary of reported CD44 and STAT3 interactions within the TME is visually presented in **Figure 3**. Collectively, these data may inspire a new wave of clinical investigation that will provide important insights into the clinical benefits of eliminating immunosuppression and preventing therapy resistance of ovarian cancer by targeting the CD44/STAT3 axis in different TME resident cell types.

## CD44 and STAT3 in Ovarian Cancer Metabolism

Metabolism rewiring is a hallmark of cancer progression across numerous tumor models (204). Below, we discuss potential roles for CD44 and STAT3 in different aspects of metabolism switch in cancer cells that support tumor progression. Specifically, we highlight available findings on potential CD44 and STAT3 cooperation in promoting glycolysis or lipid catabolism.

### CD44 and STAT3 in Glycolysis

Enhanced glycolysis, known as the Warburg effect (WE), fulfils high energy demand and provides metabolic intermediates involved in synthesis of building blocks for cancer growth, progression, and chemoresistance. WE is widely believed to predominate in many cancers, including ovarian (205, 206). CD44 is suggested to regulate glucose metabolism by switching metabolic pathway to elevated glycolysis and increasing energy production via interacting with the glycolytic enzyme PKM2 in cells either with p53 deficiency or under hypoxic conditions. Depletion of CD44 sensitizes colorectal cancer cells to chemotherapeutics potentially due to accumulated cellular reactive oxygen species (ROS) (207). Furthermore, overexpression of CD44 enhances glycolytic activity in the highly aggressive prostate small cell neuroendocrine carcinoma (SCNC) via PFKFB4 upregulation, whereas knockdown of CD44 by RNA interference increases the sensitivity of SCNC cells to carboplatin (208). In breast cancer, the dependency on glycolysis for CD44+ CSCs has been demonstrated by the enrichment of essential enzymes of glycolysis for maintaining cancer stem-like properties under hypoxic conditions (209, 210). The mechanistic study performed by Nam et al. further confirmed CD44-mediated regulation of glycolysis in breast cancer cells via LDH1 isoform upregulation by the CD44-activated c-Src/Akt/LKB1/AMPKα signaling pathway (211). The activation of HIF-1α associated signaling cascades by CD44 also contributes to enhanced glycolytic phenotype (212). Of interest, CD44+ ovarian CSCs show preference for glycolysis as well, which indicates an important role for CD44 in ovarian cancer stemness maintenance via metabolism regulation (213). Likewise, many laboratories have shown that STAT3 promotes glycolysis by upregulating HIF-1α and consequently inducing glycolytic gene (e.g. PKM2) expression across various tumors (214–216). The ability of both CD44 and STAT3 to activate HIF-1α associated pathways may suggest their concomitant impact on glucose metabolism during ovarian cancer progression.

### CD44 and STAT3 in Lipid Metabolism

In addition to the effects of elevated glycolysis on cancer cells, mounting evidence has recently demonstrated the importance of lipid metabolism in promoting cancer aggressiveness, metastasis, and chemoresistance (217–219). First, facilitating exogeneous fatty acid uptake from visceral adipocytes through elevated fatty acid receptor CD36 on ovarian cancer cells contribute to tumor progression and peritoneal metastatic module formation (220). Second, ovarian cancer reprogramming towards upregulated lipogenesis supports tumor growth and metastasis in the omental and ascitic microenvironments (221). Finally, catabolism of fatty acids mainly through fatty acid β-oxidation (FAO) promotes ovarian cancer malignant transformation by overexpression of CPT1A, an isoform of CPT1, which is a rate limiting enzyme involved in FAO (222).

Intriguingly, CD44 signaling has been implicated in lipid metabolism during malignant progression. Inhibition of FASN and ACLY, the key enzymes regulating de novo lipid synthesis, significantly downregulates CD44 expression and disrupts the CD44/c-MET complex formation, which suppresses the activation of downstream Src-mediated signaling that modulates cell proliferation and invasion (223). However, despite the reliance of CD44+ CSCs on glycolysis, recently emerged evidence demonstrates CSC tendency to rely on the energy-efficient oxidative phosphorylation (OXPHOS) route as well. The CD36 overexpressing CD44+ cells possess an increased metastasis-initiating potential, which is highly dependent on the absorbed fatty acids as an energy source compared to the low CD36 non-metastatic counterparts (224). Also, CD44+ breast CSCs not only show high lipid droplet accumulation (225), but also require FAO for maintenance of self-renewal and chemoresistance through JAK/STAT3 mediated induction of CPT1B, another isoform of CPT1, as recently demonstrated by our group (226). Similarly, CD44+ CSCs derived from ovarian cancer patients show both upregulated glucose uptake and the expression of key genes associated with OXPHOS and FAO (227). The contradictory findings about the metabolism state of CD44+ CSCs may be ascribed to the existence of differential subpopulations of CD44+ cells, which display highly heterogenous gene expression profiles that determine metabolic preference and, therefore, cell fate. Understanding the metabolic characteristics associated with therapy resistance and uncovering the underpinning mechanisms, such as CD44 and STAT3 crosstalk, will provide insight into novel therapeutic interventions to overcome ovarian cancer chemoresistance.

## CD44 Targeting for Cancer Therapy

Targeted therapies are intended to specifically inhibit abnormally activated pathways within cancer cells and represent a better and more precise treatment option than conventional chemotherapy. While CD44 expression is almost undetectable on normal ovarian surface epithelium (47, 228, 229), the majority of epithelial ovarian carcinomas demonstrate high CD44 levels (38–40), which is correlated with disease progression, cancer stemness and resistance to therapy (44, 48, 55). As discussed above, the multifunctional roles of CD44 in dynamic interactions between tumors and the TME, as well as in the regulation of cancer metastasis, stemness, and chemoresistance make CD44 an attractive therapeutic target to improve clinical outcomes for ovarian cancer patients by sensitizing them to chemotherapy. Below, we review different approaches to block CD44 signaling in pre-clinical studies and highlight the outcomes of different CD44 targeting clinical trials, as well as provide additional evidence for the potential benefits of dual targeting of CD44 and STAT3.

### CD44 Targeting in Pre-Clinical Studies

The main types of therapeutics that are aimed to target CD44 in tumors in both preclinical and clinical trials are neutralizing monoclonal antibodies (Mabs), HA-conjugates, and peptide mimetics (12). CD44-targeting antibodies showed a significant anti-tumor effect in xenograft pancreatic and squamous cell carcinoma models, as well as in acute myeloid leukemia (AML) (79, 230, 231). Moreover, a recombinant humanized Mab, RG7356, that selectively recognizes the HA-binding region of all CD44 isoforms, demonstrated promising results in pre-clinical studies by selectively killing leukemic B cells without affecting the viability of normal B cells in a chronic lymphocytic leukemia (CML) model (232), indicating a potential for Mabs for cancer treatment in clinic. As an alternative strategy to antibodies, HA-conjugated chemotherapeutics were formulated to specifically target tumor cells for more precise action and side-effect minimization. HA-conjugated paclitaxel has been shown to selectively bind CD44+ tumor cells and block cancer cell line proliferation *in vitro* of numerous cancer types, including ovarian (233, 234). Finally, specific peptides that mainly target HA-CD44 interaction have been reported. Specifically, CD44-binding peptide A5G27 showed significant inhibitory effect on tumor growth and metastasis in a mouse melanoma model (235), while another CD44-targeting peptide, A6, inhibited migration, invasion, and metastatic potential of prostate, breast and ovarian cancer cells (236–238). Taken together, numerous studies demonstrate the beneficial effects of CD44 inhibition on tumor progression. This supports CD44 as a promising clinical target for the development of novel ovarian cancer therapeutics.

### Combined CD44 and STAT3 Inhibition

Relevant to the scope of this review, several studies have demonstrated beneficial effects of combined CD44 and STAT3 signaling downregulation across different cancer models, including ovarian cancer. Gemini vitamin D analogue BXL0124 inhibits CD44-STAT3-mediated breast cancer invasion and metastasis by decreasing CD44 expression and STAT3 activation, as well as preventing CD44 binding to JAK2 and STAT3 in the cytoplasm (80). Likewise, Zerumbone, a monocyclic natural anti-inflammatory and antioxidant agent, suppresses EGF-induced CD44 expression through STAT3 pathway inhibition in breast cancer cell lines, while combined STAT3 and NF-kB inhibition with curcumin and epigallocatechin gallate decreases the CD44+ breast CSC population (88, 239). More importantly, in ovarian cancer cells, a natural compound from *Tripterygium wilfordii*, Celastrol, promotes apoptosis by decreasing CD44 expression and STAT3 phosphorylation (240). Additionally, ovarian cancer cell stemness is reduced by the FK506-binding protein like (FKBPL) peptide via inhibiting the CD44/STAT3 signaling axis (241) and the medicinal herb corilagin sensitizes human ovarian cancer cell lines to chemotherapy by glycolysis inhibition via downregulation of both CD44 and STAT3 expression (242). Finally, an orally administered small molecule STAT3 inhibitor Napabucasin, which is currently being tested in several clinical trials against various cancer models (243), have been shown to decrease both STAT3 activation and CD44 expression in biliary tract cancer cells (244). Collectively, these observations indicate that CD44 and STAT3 molecular cooperation deserves further attention and may be a promising clinical target to develop more effective therapeutics for the treatment of ovarian tumors. Given that CD44/STAT3 axis is involved in cancer progression and therapy resistance, we hypothesize that combinatorial administration of the most promising targeting agents, such as A6 blocking peptide against CD44 and Napabucasin against STAT3, can be beneficial for ovarian cancer patients in advanced disease stage and deserves further investigation.

### CD44 Targeting in Clinical Trials

To date, several clinical trials have tested different CD44 targeting agents to generate a cancer-specific drug delivery system in the clinic. The outcomes of different trials analyzing CD44-targeting agents are summarized in **Table 2** (245–254).

**Table 2:** Clinical studies investigated the efficacy of CD44 targeting.

Ref.	Phase	Cancer type	Target isoform	Drug name/conjugation	Outcome	Adverse effects
(245)	Phase I	Head and neck squamous cell carcinoma	CD44v6	Bivatuzumab	Fatal drug-related adverse event occurred, termination	Skin-related adverse events
(245)	Phase I	Squamous cell carcinoma	CD44v6	Bivatuzumab	14% (2 of 7) patients had stable disease	One drug-related fatality and various grade skin reactions
(246)	Phase I	Head and neck squamous cell carcinoma	CD44v6	Bivatuzumab	Fatal drug-related adverse event occurred, termination	Skin-related adverse events
(247)	Phase II	5-FU-resistant metastatic colorectal cancer	CD44	HA-Irinotecan	17% partial response and 50% stable disease	Dose-limiting toxicity
(248)	Phase IIa	Extensive-Stage Small Cell Lung Cancer	CD44	HA-Irinotecan	No difference in survival outcomes	Not reported
(249)	Phase I	CD44-expressing solid tumors	CD44	RG7356	21% patients had stable disease	Fever, headache and fatigue
(250)	Phase I	Relapsed/refractory acute myeloid leukemia	CD44	RG7356	One patient had stable disease; 2 patients complete response with incomplete platelet recovery or partial response	Dose-limiting toxicity and moderate adverse events
(251)	Phase I	Advanced gynecologic cancer	CD44	A6	The safety outcome in this Phase1b gynecologic cancer trial was excellent and showed no specific toxicity profile	No systemic drug-related adverse events
(252)	Phase II	epithelial ovarian, fallopian tube, or primary peritoneal cancer	CD44	A6	36% (4/11) of patients had disease stable	No systemic drug-related adverse events
(253)	Phase II	Persistent or recurrent epithelial ovarian, fallopian tube, or primary peritoneal carcinoma	CD44	A6	6.5% were progression free for at least 6 months 18.5% (2/27); with CD44+ expression	One fatal drug-related event
(254)	Phase I	Advanced ovarian epithelial cancer	CD44	SPL-108	Phase I: health volunteers no systemic adverse events; phase Ib: showed self-limited, mild or moderate adverse events with several subjects showing stable disease	Not reported

Thus far, clinical trials of targeted CD44 therapies have focused on either humanized or immunoconjugated Mabs, such as RG7356 and Bivatuzumab, respectively, HA-conjugated chemotherapeutics, or CD44-targeting peptide A6 also known as SPL-108. As seen in **Table 2**, Bivatuzumab, a CD44-specific Mab conjugated with mertansine, demonstrates severe adverse effects related to serious skin toxicity with fatal outcomes (245, 246). Humanized RG7356 antibody shows mild adverse effects and was well tolerated in advanced solid malignancy and AML trials, however solid tumor clinical response showed only 21% efficacy (249, 250). As an alternative to antibodies, a specific drug formulation covalently attached to HA has been tested. HA-bound irinotecan, a topoisomerase-1 inhibitor, utilizes the unique biologic properties of HA to specifically target CD44-expressing solid tumor tissues and initially improved median progression-free survival and clinical outcomes of colorectal and small cell lung cancer patients in two different trials (247, 248). However, another HA-irinotecan trial demonstrated statistically significant improvement in progression-free survival of metastatic colorectal cancer patients in Phase II trials but could not reproduce these results in Phase III studies (255), indicating that further trials are still needed. Finally, the aforementioned A6/SPL-108 peptide showed a promise in several ovarian cancer trials. Continuous daily consumption was well tolerated without any dose-limiting toxicity and time to clinical disease progression was significantly increased in the EOC patient population (251, 252). However, A6/SPL-108 had minimal activity in patients with persistent or recurrent disease (253), indicating a need for further optimization or, potentially, incorporating dual inhibition of CD44 and its relevant interaction partners, such as STAT3.

## Conclusions and Future Directions

Ovarian cancer is one of the leading causes of death due to malignancy among women worldwide. Frequent metastasis due to advanced stage at the time of diagnosis, disease recurrence, and chemoresistance are major hurdles in the clinic and there is an urgent need to identify suitable molecular targets that drive the disease and design specific therapeutic strategies to circumvent these problems. CD44 and STAT3 cooperate at multiple levels in both malignant and the normal cells in the tumor microenvironment, leading to cancer progression and resistance to therapies. In addition, a critical role of CD44/STAT3 interaction in inducing immunosuppression has been highlighted. These findings taken together suggest that targeting CD44-STAT3 axis effectively can be an advantageous strategy for treating ovarian cancer.

While we did not discuss the recent advances of PARP inhibitors, accumulating clinical data indicate they can have excellent responses in a subset of ovarian patients with BRCA 1, BRCA2 or other homologous recombination alterations (256). However, PARP inhibitor-resistance is common (257) and PARP inhibitor-treatment leads to activation of STAT3 (258), which likely increases the expression of CD44. The possibility of targeting CD44/STAT3 axis to boost the antitumor efficacies of PARP inhibitors and overcome PARP inhibitor resistance may lead to better treatment for ovarian cancer patients.

## Author Contributions

HY and LR-R conceived and supervised the manuscript writing. AM created the figures, found and summarized relevant literature findings for each chapter and wrote the final manuscript. P-CL created the tables and summarized relevant literature findings for most of the manuscript parts. QZ prepared and summarized relevant findings for *CD44 and STAT3 in Ovarian Cancer Metabolism* section of the manuscript under the guidance of Y-JL. CZ prepared and summarized relevant findings for *CD44 and STAT3 in Regulatory T Cells* and *CD44 and STAT3 in Regulatory B Cells* sections of the manuscript. All authors contributed to the article and approved the submitted version.

## Funding

This work is supported by the Markel-Friedman Accelerator Fund.

## Conflict of Interest

The authors declare that the research was conducted in the absence of any commercial or financial relationships that could be construed as a potential conflict of interest.

The reviewer AS declared a shared affiliation, with no collaboration, with the authors to the handling editor at the time of review.
